# Immune checkpoint inhibitor-related pneumonitis: research advances in prediction and management

**DOI:** 10.3389/fimmu.2024.1266850

**Published:** 2024-02-15

**Authors:** Mei-Xi Lin, Dan Zang, Chen-Guang Liu, Xu Han, Jun Chen

**Affiliations:** Department of Oncology, The Second Hospital of Dalian Medical University, Dalian, China

**Keywords:** malignancy, immune checkpoint inhibitor-related pneumonitis, diagnosis, treatment, prediction

## Abstract

The advent of immune-checkpoint inhibitors (ICIs) has revolutionized the treatment of malignant solid tumors in the last decade, producing lasting benefits in a subset of patients. However, unattended excessive immune responses may lead to immune-related adverse events (irAEs). IrAEs can manifest in different organs within the body, with pulmonary toxicity commonly referred to as immune checkpoint inhibitor-related pneumonitis (CIP). The CIP incidence remains high and is anticipated to rise further as the therapeutic indications for ICIs expand to encompass a wider range of malignancies. The diagnosis and treatment of CIP is difficult due to the large individual differences in its pathogenesis and severity, and severe CIP often leads to a poor prognosis for patients. This review summarizes the current state of clinical research on the incidence, risk factors, predictive biomarkers, diagnosis, and treatment for CIP, and we address future directions for the prevention and accurate prediction of CIP.

## 1 Introduction

Immune checkpoint inhibitors (ICIs) target tumor cells through the immune system and have innovated the treatment of many advanced malignancies. These inhibitors have received approval for diverse indications, such as programmed cell death receptor 1 (PD-1) inhibitors (e.g., Pembrolizumab), cytotoxic T-lymphocyte-associated antigen 4 (CTLA-4) inhibitors (e.g., Ipilimumab), and programmed cell death ligand 1 (PD-L1) inhibitors (e.g., Atezolizumab). However, inhibition of the immune checkpoint/receptor axis can disturb the normal mechanisms involved in immune tolerance and lead to immune-related adverse events (irAEs) (1). Despite the significant clinical benefits of ICIs, irAEs often carry the risk of discontinuation, drug switching, and patient deterioration. In comparison with chemotherapy-related adverse events, irAEs generally depict a delayed onset and longer duration, and effective management relies on early recognition and timely intervention, including discontinuation, immunosuppression, and/or immunomodulatory strategies (2). However, severe irAEs are sometimes fatal, and among them, immune checkpoint inhibitor-related pneumonitis (CIP) can lead to widespread respiratory symptoms and parenchymal abnormalities, and consequently result in respiratory failure, even death. CIP is termed a highly common cause of fatality associated with anti-PD-1/PD-L1 immunotherapy and accounts for approximately 35% of causes of fatalities (3, 4). Although clinical trials have shown a rare incidence of CIP (usually <5%) (5), real-world studies have shown that its incidence ranges from 5% to 19% in lung cancer cohorts (6, 7). Prior research has indicated that individuals with irAEs have significantly longer overall survival (OS) and progression-free survival (PFS) than individuals without irAEs. However, there was no significant correlation between CIP and the efficacy of immunotherapy in subgroup analysis (8, 9). In contrast, one study observed improved ICI efficacy of grade 1-2 CIP, whereas no correlation was observed between grade 3-4 CIP and the efficacy of ICIs (10). A meta-analysis suggested that adverse effects in other organs, such as endocrine and skin, were associated with benefits in OS analysis, while CIP was significantly heterogeneous (11). Diagnosing and treating CIP can be challenging due to its wide range of symptoms, ranging from asymptomatic cases to severe acute respiratory distress syndrome. Furthermore, our understanding of the pathogenesis underlying CIP remains limited. For the occurrence of CIP, immunotherapeutic drugs need to be suspended, and corticosteroid therapy, as well as empirical anti-infective therapy, is given. However, this also leads to delays in anti-tumor therapy. Therefore, it is necessary to identify risk factors and explore more effective biomarkers to prevent the occurrence of CIP and to better manage the adverse effects that have already occurred. Herein, we combed through ideas for CIP management based on clinical experience, and the mechanism, incidence, risk factors, diagnosis, treatment, and predictive biomarkers of CIP are narratively summarized, aiming to analyze the clinical features of CIP and guide the management of CIP patients.

## 2 Mechanism of CIP

Normally, when non-self cells such as tumor cells are detected, antigen-presenting cells (APCs) such as dendritic cells and macrophages internalize and deliver tumor antigens via the major histocompatibility complex (MHC). The MHC then binds to T-cell receptors. When additional synergistic interactions occur, such as those involving the CD28 receptor, T cells are fully activated, initiating a cascade of cytotoxic responses aimed at eliminating the tumor (12, 13). During T cell activation, there is also an upregulation of various inhibitory receptors that serve as immune checkpoints. Immune checkpoint proteins including CTLA-4 and PD-1 play a key role by initiating various pathways to inhibit T cell function. PD-1 expression has been observed on a variety of immune cells, including B cells, T cells, and natural killer (NK) cells. In addition, PD-1 binding to the ligands PD-L1 and PD-L2 has been observed to inhibit previously activated T cells in peripheral tissues. CTLA-4 functions by competing with the T cell fibrinolytic receptor CD28 for binding to T cell fibrinolytic factors. As a result, it reduces interleukin-2 (IL-2) production and T-cell proliferation. The tightly regulated signaling of CTLA-4 and PD-1 plays a critical role in maintaining self-tolerance within the immune system. However, tumor cells can use these pathways to evade the immune response and create a growth-promoting microenvironment (14). ICIs primarily target two key immune checkpoint pathways, CTLA-4 and PD-1, which are commonly involved in down-regulating T cell activation and effector functions. By inhibiting these pathways, ICIs can enhance T cell-mediated anti-tumor immune responses without the typical limitations of these checkpoints (15).

**Figure 1:**
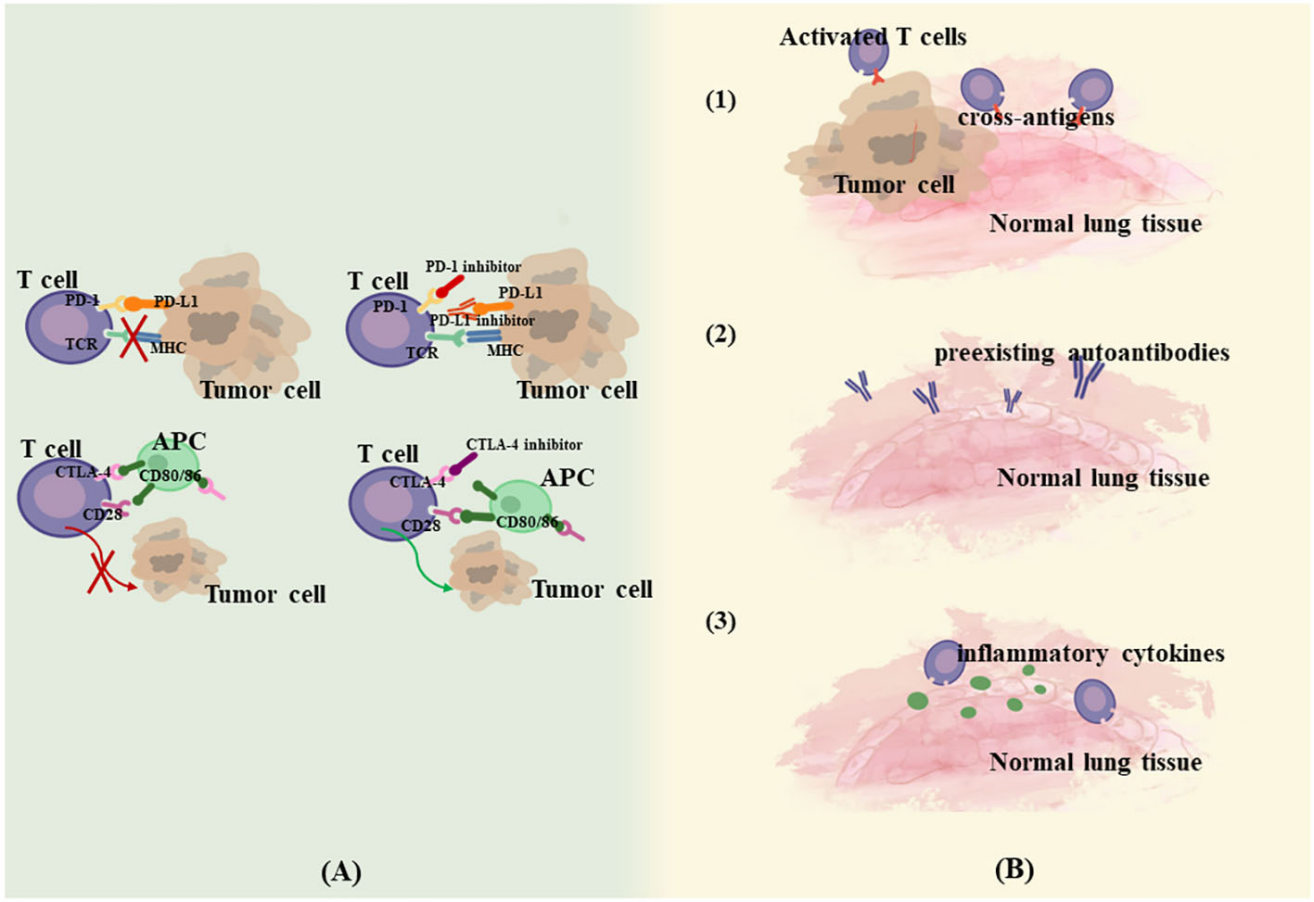
Mechanism of CIP. **(A)**. Anti-tumor mechanism of ICIs. **(B)**. Possible mechanism of CIP. (1) Enhanced targeted T-cell activity attack cross-antigens shared between tumors and normal lung tissue. (2) Increased levels of preexisting autoantibodies. (3) Increased levels of inflammatory cytokines.

Interference with the immune checkpoint pathway is the primary mechanism for enhancing the immune response against tumor cells, but this pathway has also been implicated in the emergence of various irAEs. IrAEs are coordinated predominantly by T-cells, and significant infiltration of CD4+ and CD8+ cells can be observed in conjunction with the onset of irAEs (16). Additionally, an ensemble of immune cells and mediators, including B cells, granulocytes, and cytokines, are also implicated in this process (17). This heightened immune activity culminates in reactions that resemble autoimmune responses, which are characteristic of irAEs. A variety of mechanisms have been suggested to be involved in the development of irAEs. Postow et al. (18) proposed four potential mechanisms for irAEs. (1) Enhanced targeted T-cell activity can attack cross-antigens shared between tumors and normal lung tissue, leading to off-target toxicity. Multiple experiments examined significant CD4+ T lymphocyte and CD8+ T lymphocyte increases in lung tissues and BAL of CIP patients, reflecting a lymphocyte-mediated hyperimmune response (19–21). (2) Increased levels of preexisting autoantibodies. Osorio JC et al. demonstrated that patients treated with anti-PD-1 therapy may develop thyroid dysfunction if antithyroid antibodies are present in the body, and a possible mechanism for this is that anti-PD-1 therapy, in addition to mediating T-cell immunity, modulates humoral immunity and enhances preexisting antithyroid antibodies (22). (3) Increased levels of inflammatory cytokines. (4) Direct binding of ICIs to normal tissues. For example, anti-CTLA-4 antibodies can directly bind to CTLA-4 expressed on the pituitary gland, thus triggering pituitary inflammation (23). According to Zhai et al., the mechanism of CIP may be more relevant to the first three theories since PD-1/PD-L1 is expressed predominantly in immune cells and virtually none in normal lung tissue (24) (**Figure 1**). Currently, the key biological mechanisms underlying CIP are poorly understood, and it is difficult to determine whether they are caused by disturbances in the local immune response, hypersensitivity, direct drug effects, or a combination of factors. The episodic, unpredictable, and relatively rare nature of CIP makes it difficult to study systematically, and the mechanisms may be different in patients with steroid-refractory. The combination of mechanistic biochemical *in vitro* studies, construction of animal models, and the use of human specimens in translational research may contribute to a better understanding of the biological mechanism. Reference can also be made to existing knowledge of the biology of lung diseases such as interstitial lung disease (ILD) and drug-associated pneumonitis.

## 3 Real-world incidence of CIP

The incidence of CIP in daily clinical practice and the identification of patients at risk of these potentially life-threatening irAEs are critical to addressing the more at-risk patients with lung cancer. Real-world data (RWD) has become increasingly important in this field considering the accelerated approval of cancer immunotherapies. Initially observed at an incidence of 3-5% in clinical trials, CIP was observed to be more common in the real world (25). A study involving 315 patients with lung cancer treated primarily with nivolumab or pembrolizumab depicted a 9.5% incidence of CIP. The median time to diagnosis was 52.5 days, with most patients with CIP depicting a high severity of the disease. Additionally, during the ongoing CIP treatment, eight patients (27%) unfortunately succumbed to the condition. Chao et al. (26) reviewed a study in which CIP occurred in 20 of 164 patients with non-small cell lung cancer (NSCLC) treated with ICIs, accounting for 12.2%. Naidoo J et al. (7) counted 915 patients with various types of advanced solid tumors treated with anti-PD-1/PD-L1 antibodies, 43 of whom suffered from varying degrees of pneumonitis, with a higher prevalence of pneumonitis with combination immunotherapy compared with monotherapy (10% vs. 3%). Suzuki Y et al. reported a prospective study that specifically assessed the incidence and risk factors for CIP in clinical practice in 138 patients with advanced NSCLC who were mainly treated with nivolumab as second or later line. The incidence of CIP was found to be 14.5% (with approximately 6% of ≥ grade 3 events occurring earlier than low-grade events), which is much higher than commonly described in clinical trials or meta-analyses (27). It has been reported (28–30) that although mild CIP can occur more than six months after the start of ICIs, most of the severe CIP have an earlier onset. Moreover, 10-20% of CIP end up being incurable or even leading to death. Thus, CIP occurs far more often than is commonly recognized and is prone to serious adverse outcomes, requiring strict monitoring during drug administration (**Table 1**).

**Table 1:** The incidence of CIP in real world.

Total number	CIP patients	Incidence rate	Median onset time	Cancer type	ICIs treatment	Refs
101	22	21.78%	4.5 months	lung cancer	anti-PD-1	(1)
315	30	9.50%	1.8 months	lung cancer	anti-PD-1	(3)
270	6	2.22%	not mentioned	lung cancer	anti-PD-1 (89.3%) anti-PD-L1 (10.7%)	(8)
559	23	4.11%	not mentioned	lung cancer	anti-PD-1	(9)
71	22	30.90%	not mentioned	lung cancer	anti-PD-1 (85.9%) anti-PD-L1 (14.1%)	(10)
164	20	12.20%	2.9 months	lung cancer	anti-PD-1,anti-PD-L1	(26)
170	27	15.88%	1.2 months	lung cancer	anti-PD-1	(30)
204	38	18.63%	6.3 months	lung cancer	anti-PD-1 (91.2%) other(10.8%) Combination ICIs(30.9%)	(28)
1826	64	3.50%	2.3 months	lung cancer (75%) melanoma (20.3%) other types (4.7%)	anti-CTLA-4 (6.9%) anti-PD-1 (79.3%) anti-PD-L1 (13.8%)	(29)
406	16	3.94%	Not mentioned	renal cell carcinoma	anti-PD-1	(31)
138	20	14.50%	1.7 months	lung cancer	anti-PD-1	(27)
71	1	1.41%	1.4 months	melanoma	anti-CTLA-4	(32)

## 4 Risk factors of CIP

**Figure 2:**
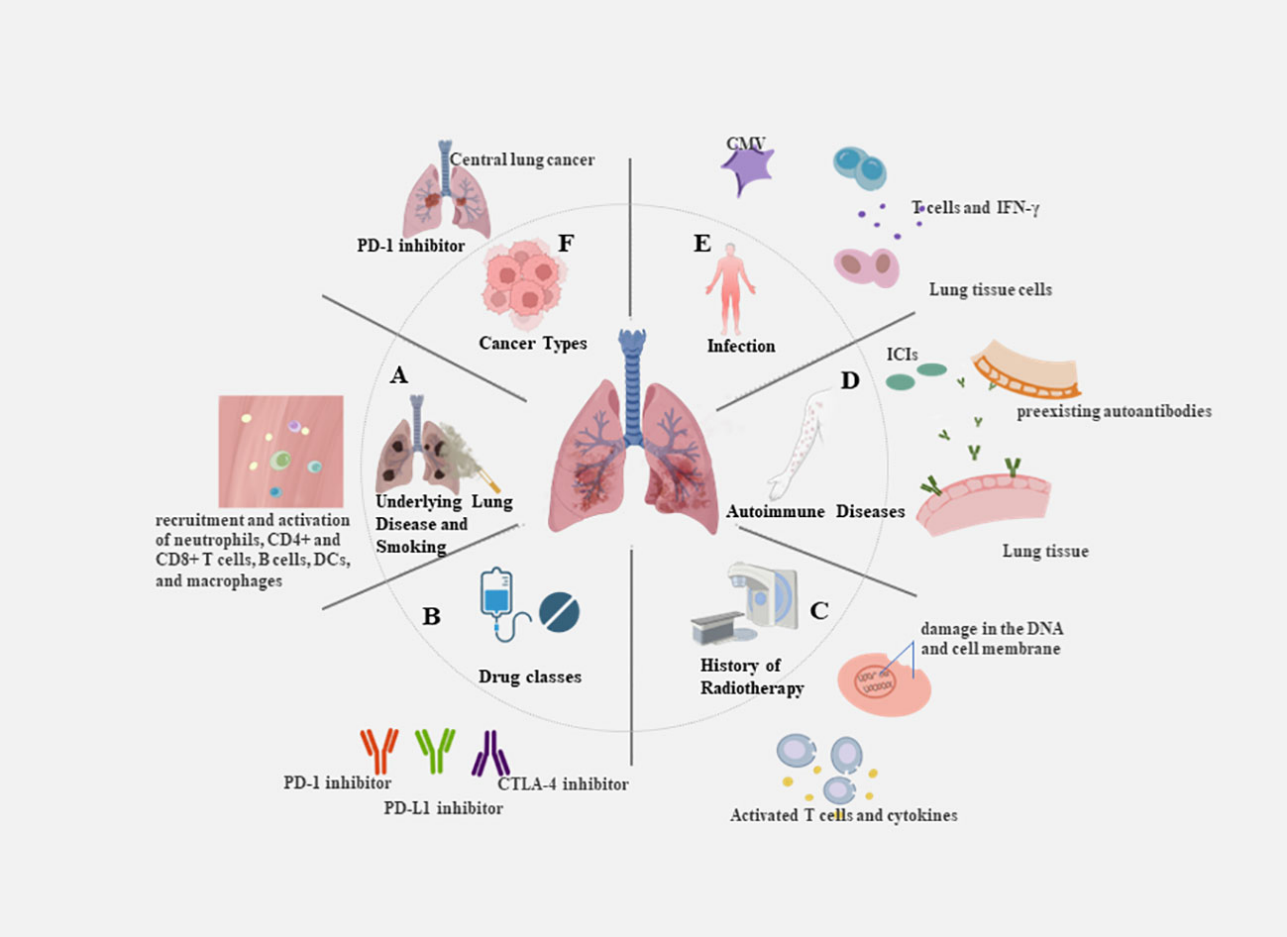
Risk factors for the occurrence of CIP. **(A)** Underlying lung disease and smoking: e.g. The inflammatory microenvironment in COPD patients is accompanied by the recruitment and activation of neutrophils, CD4+ and CD8+ T cells, B cells, dendritic cells, and macrophages. **(B)** Drug classes: e.g. PD-1 inhibitors revealed an increase in the incidence of pneumonia of any grade in comparison with PD-L1 inhibitors. **(C)** History of radiotherapy: e.g. The combination of DNA damage caused by radiotherapy and the reactivation of T cells by immunotherapy results in the release of large amounts of cytokines. **(D)** Autoimmune diseases:e.g. Patients treated with ICIs modulates humoral immunity and enhances preexisting autoantibodies. **(E)** Infection: e.g. CMV infection or reactivation can lead to severe disease in the absence of an effective immune response, with increased CD8+ T cell sensitivity and elevated levels of circulating IFN-γ. **(F)** Cancer types: e.g. Squamous cell carcinomas are predominantly central lung cancers that are more prone to causing obstructive pneumonia.

The uncontrolled activation and proliferation of T cells can result in an excessive release of cytokines, triggering an excessive immune response and contributing to the development of CIP. With combination chemotherapy and ICIs now being first-line treatments for many malignant solid tumors, the need to understand potential increased risk of CIP is even more critical. Any delay in the prompt treatment of CIP patients may result in exacerbation of the disease. Because symptoms are not specific, many early CIP patients are overlooked and lead to poor outcomes. Therefore, it is critical to screen people at high risk of CIP and identify predictive biomarkers to enable its early identification. There is no standardized predictive model for CIP, and the identification of various risk factors comes mainly from summarizing clinical practice. The characteristics of the patient’s primary disease, physical status, and treatment modality may influence the development of CIP. Potential severity of CIP emphasizes the need to detect baseline predictive factors contributing to the assessment of the individual risk-benefit ratio of a treatment. It is essential to determine biomarkers that possess key advantages, such as being easy to collect, minimally invasive, and reproducible for application in actual clinical settings (33) (**Figure 2**).

### 4.1 Underlying health status

#### 4.1.1 Underlying lung disease and smoking history

The development of CIP results in damage to the lung parenchyma, and disease of the underlying “soil” of the lungs may result in a weakened ability to resist damage. Heavy smoking affects the lungs prior to treatment with ICIs, leading to chronic respiratory diseases such as atelectasis and chronic obstructive pulmonary disease. Treatment of ICIs in patients with poor lung conditions can easily lead to CIP. Atchley et al. (3) assessed the records of lung cancer patients exposed to ICIs monotherapy or combination therapy at six centers in North Carolina (January 2004-July 2017). The research found that the development of CIP was linked independently with baseline fibrosis on chest CT scan, and a composite of obstructive lung disease was independently associated. Chao et al. (26) performed a regression analysis of NSCLC patients using PD-1/PD-L1 and found that the presence of chronic obstructive pulmonary disease (COPD) and PD-L1 expression ≥ 50% were linked to an increased CIP prevalence independently. This may be due to the fact that the inflammatory microenvironment in COPD patients differs from other patients in the presence of chronic inflammation in tissues accompanied by the recruitment and activation of neutrophils, CD4+ and CD8+ T cells, B cells, dendritic cells, and macrophages (34). The activated T cells increase in tumor and healthy lung tissues and result in modulating the inflammatory response to CIP (18, 35). Research by Zhang et al. also observed a higher grade and incidence of CIP in individuals with pre-existing ILD (36). In the research of Pérol et al. (37), the risk of CIP was also elevated in real-world individuals with a previous history of noninfectious pneumonia. The presence of ILD also has an impact on the time to onset of CIP, with studies noting that the median time to onset of pneumonia from initiation of anti-PD-1 antibody therapy was 1.3 months (range 0.3 to 2.1 months) for patients with NSCLC with preexisting ILD and 2.3 months (range 0.2 to 14.6 months) for those without preexisting ILD (38). However, there are also experimental results that contradict the above conclusions. Horiuchi et al. retrospectively evaluated 209 patients with NSClC, malignant melanoma, renal cell carcinoma (RCC), and gastric cancer (GC) treated with anti-PD-1/PD-L1. Multifactorial logistic analyses of baseline characteristics showed that a history of cigarette smoking was the only significant predictor of CIP, whereas no statistically significant associations were detected between a history or radiologic features of preexisting ILD and CIP. Smoking history is an independent influence on CIP, and the column-line graph shows that smoking history is the most influential prognostic factor (39). Multiple retrospective clinical studies reveal the correlation between smoking and the development of CIP. Smoking history is an independent influence and the most influential prognostic factor in CIP (40, 41). Studies also showed that clinical outcomes of CIP worsen more frequently in patients with a history of smoking (7). The retrospective study by Okada et al. showed that ≥ 50 pack-years was an independent risk factor associated with all levels of CIP (42). However, a history of smoking has also been suggested as a prognostic marker for ICIs treatment. Studies have shown that smoking-induced DNA damage may benefit ICIs treatment, and lung cancer patients who smoked for more than 20 pack-years exhibited genetic mutations associated with a favorable response to ICIs therapy (43). In summary, smoking is beneficial to the efficacy of ICIs and is also a risk factor for CIP, depending on the frequency of smoking. This result suggests that a history of smoking and a quantitative assessment of smoking should be considered when treating lung cancer with ICIs. It is evident that a detailed understanding of the patient’s lung disease history should be obtained before treatment with ICIs and more rigorous monitoring should be provided for this population; however, most previous studies have been limited to small samples, and the extent to which different underlying lung diseases contribute to CIP needs to be classified in further prospective studies.

#### 4.1.2 Cancer types and drug classes

The incidence of CIP varies with cancer types and treatment modalities. A meta-analysis of trials involving 20 anti-PD-1 treatments for melanoma, NSCLC, and RCC indicated the heightened occurrence of all-grade and grade ≥ 3 pneumonia in individuals with NSCLC than in individuals with melanoma. Compared with melanoma, patients with RCC had a higher incidence of all-grade pneumonia but a lower incidence of grade ≥ 3 pneumonia (31). It is unclear why NSCLC may be associated with more pneumonia and treatment-related deaths, but several hypotheses seem plausible, including preexisting adverse lung conditions and prior exposures to medications associated with ILD, including paclitaxel, epidermal growth factor receptor tyrosine kinase inhibitors, and gemcitabine. Several studies have shown (44–47) PD-1 inhibitors to have lower rates of irAEs than CTLA-4 inhibitors, while combination therapy depicted higher rates of irAEs than monotherapy. A concurrent analysis of 19 trials of PD-1 and PD-L1 for NSCLC found that PD-1 inhibitors revealed an increase in the incidence of pneumonia of any grade and grade ≥ 3 in comparison with PD-L1 inhibitors. Untreated patients also had an increased incidence of pneumonia in comparison with patients who had been treated previously (48). Chen X et al. (49) found an increased risk of CIP with ICIs in combination with chemotherapy compared to chemotherapy. However, the risk was still lower than with ICIs alone or double-free combination therapy. This may be attributed, in part, to the cytotoxic effects of chemotherapeutic agents, which can lead to immunosuppression. Additionally, the use of glucocorticoids as pre-treatment for chemotherapy may contribute to immune system suppression. Glucocorticoids are also used to treat underlying lung diseases including asthma and COPD. In addition, anti-angiogenic drugs (e.g., bevacizumab) decrease pulmonary exudation and vascular permeability, potentially aiding in the recovery of early-stage pneumonia (50). This research exhibited that squamous cell carcinoma of the lung may be a risk factor for pneumonia, and similarly, during the assessment of 87 patients with CIP, Lin X et al. found that squamous cell carcinoma subtype and ICIs monotherapy were independently and notably associated with the development of CIP (51). This correlation can be attributed to the fact that obstructive pneumonia can elevate the risk of CIP, and squamous cell carcinomas are predominantly central lung cancers that are more prone to causing obstructive pneumonia. In contrast, Pérol et al. mentioned that the only disease characteristic linked to the risk of pneumonitis is adenocarcinoma histologic subtype (37). The use of treatment combinations in a later study, ethnicity, and different smoking habits might explain these opposite findings.

#### 4.1.3 History of radiotherapy

Radiotherapy provides excellent local control of tumor growth. However, it is important to note that radiotherapy can also exert various immunomodulatory effects. Radiotherapy can be linked to inducing damage in the DNA and cell membrane and is also involved in increasing reactive oxygen species (ROS). It further activates transcription factors and signaling pathways, modulates the immune phenotype and immunogenicity of tumor cells, restores anti-tumor T-cell responses in the tumor microenvironment, and increases tumor antigen release while improving antigen presentation and T-cell infiltration (52, 53). Many pro-inflammatory cytokines and chemokines are systemically increased in immune cells and tumor tissues after radiotherapy, which may account for the nonspecific eradication of distant tumors and metastases (54). Although ICIs can overcome T cell suppression, T cell activation depends on the engagement of antigen receptors and activating co-stimulatory molecules expressed by mature APCs. Thus, radiotherapy increases the production and expression of tumor antigens in poorly immunogenic tumors, thereby enhancing the antitumor immune response elicited by ICIs (55). Fractionated radiotherapy combined with anti-PD-1/PD-L1 antibodies produces an effective CD8+ T-cell response and improves local tumor control and long-term survival (56). Researchs have shown that prior exposure to radiotherapy elevates the risk of developing pneumonia (38, 57). Therefore, in the real world, the advent of combined treatment modalities may increase the risks associated with treatment. Barrón F et al. (1) observed a PFS of 16.8 months versus 5.6 months and a heightened remission rate in the group treated with the combined modality of radiotherapy and anti-PD-L1 (Durvalumab) in comparison with radiotherapy and placebo in individuals with NSCLC. However, combination therapy resulted in elevating the risk of pneumonia of any grade observed in both groups, leading to treatment discontinuation. Similarly, a secondary analysis of phase I KEYNOTE-001 (58) evaluated adverse events in 97 individuals with NSCLC exposed to pembrolizumab. This analysis reported that CIP occurred in 8% (2/24) of patients who had previously undergone prior chest radiotherapy, while 1% (1/73) of patients who had not previously undergone chest radiotherapy developed CIP. In terms of the mechanism of lung injury, the combination of cellular damage caused by radiotherapy and the reactivation of T cells by immunotherapy results in the release of large amounts of cytokines. These cytokines not only directly damage lung tissues through signaling pathways such as transforming growth factor-β/drosophila mothers against decapentaplegic protein (TGF-β/Smad), tumor necrosis factor-α/nuclear factor kappa-light-chain-enhancer of activated B cells (TNF-α/NF-κB), ROS/reactive nitrogen species (RNS), and (cGMP-AMP) synthase-stimulator of interferon genes (cGAS-STING), but also induce lung injury through indirect responses such as recruitment of neutrophils, macrophages, and lymphocytes. The possible crosstalk among signaling pathways mainly involves cytokines such as IL-3, IL-6, IL-10, and IL-17; TNF-α; and TGF-β (59). The resulting data suggest that particular attention should be paid to the occurrence of CIP in individuals who received radiotherapy during treatment, and we should put strict limits on the amount of radiation to which normal lung tissue is exposed through the use of lung dose-volume histograms. Nonetheless, Atchley et al. (3) performed a large retrospective analysis, no clear correlation was found between the risk of CIP and increasing age, history of chest radiotherapy, or tumor histological type, where the association needs to be further explored.

#### 4.1.4 Autoimmune diseases

Preexisting autoimmune diseases are mostly considered contraindications to immunotherapy in the clinic due to severe immunotoxicity and the possibility of disease outbreaks. However, safety data for ICIs in patients with preexisting autoimmune diseases have been reported in several case reports and retrospective studies, which included findings of no difference in grade 3–4 irAEs in patients with or without pre-existing autoimmune disease (60, 61). Given the efficacy of ICIs for metastatic cancers, clinicians have suggested that they should be used to treat a broader population. However, the use of ICIs remains challenging, particularly with regard to the risk of causing irAEs. It is well established that ICIs treatment may trigger acute exacerbations and deterioration of autoimmune diseases. However, the safety and efficacy of ICIs in patients with pre-existing autoimmune disorders are not well documented. As a result, there is still no definitive answer regarding the safe use of ICIs in this particular patient population. Larsen et al. documented the development of anti-aminoacyl tRNA synthetase (ARS) antibody positive polymyositis at the same time as the induction of CIP by the anti-PD-1 antibody nivolumab, resulting in repeated aggravation of the pneumonitis symptoms and prolonged cycle of the treatment (62). A case report (63) documented that a male patient with antinuclear antibody-negative NSCLC was admitted to the hospital with dyspnea after receiving ICIs administration. On admission, a diagnosis of CIP was made in conjunction with imaging. Despite the administration of high-dose steroids, the patient experienced an acute exacerbation of pneumonia, along with progressive pulmonary fibrosis. Upon re-evaluation, it was discovered that the serum collected prior to the administration of ICIs contained ARS antibodies. This finding underscores the significance of reassessing pre-existing autoimmune diseases among individuals who develop CIP with atypical radiological features.

#### 4.1.5 Infection

ICIs exert their antitumor effects by restoring suppressed T-cell function. This restoration of immune function can sometimes result in an exaggerated immune response to previous infections, leading to the exacerbation of clinical symptoms linked to infection. This phenomenon is known as immune reconstitution syndrome (IRS). Cytomegalovirus (CMV) infection is prevalent in the general population, in healthy individuals, the virus and the immune system reach a homeostatic equilibrium and establish a lifelong asymptomatic latency mainly in myeloid cells (64), while in immunocompromised individuals, CMV infection or reactivation can lead to severe disease and even death in the absence of an effective immune response, with increased CD8+ T cell sensitivity and elevated levels of circulating interferon γ (IFN-γ) compared to uninfected individuals (65). Its reactivation was observed among individuals undergoing chemotherapy and radiotherapy (66). Lin X et al. (67) explored the association of CIP development with CMV infection status. Among 29 patients with grade 3–4 CIP, 12 were CMV-IgG positive, suggesting previous CMV infection. With one exception, all patients were positive for CMV PP65 antigen, implying early viral reactivation. Among them, improvement in the symptoms was observed following glucocorticoid combination antiviral therapy, except for one case with delayed antiviral therapy. It suggested that IRS induced by CMV reactivation may be crucially involved in CIP. Although CMV reactivation is uncommon in tumor patients, it is still a risk factor for CIP in patients treated with ICIs and deserves clinical attention due to the high prevalence of latent CMV infections in the population. Studies on the mechanism of CMV reactivation and the occurrence of CIP are still limited and need deeper research. In the past three years, novel coronavirus (COVID-19) pneumonia infection has affected almost everyone and has had a huge impact on the treatment of patients with malignancy. Pinato D J et al. (68) described factors associated with the development of sequelae in COVID-19 surviving oncology patients and their relationship with survival after infection, and found that 15.0% of patients had at least one COVID-19 sequelae at the time of first oncology reassessment, including 116 (49.6%) respiratory sequelae, such as chronic cough, residual dyspnea, and shortness of breath, which undoubtedly caused irreversible damage, and residual inflammatory and interstitial fibrotic lung changes are more likely to lead to CIP, while the symptoms of COVID-19-related pulmonary syndrome may be similar to the worsening of symptoms encountered during lung cancer progression (69). The similarity of clinical and imaging findings poses a greater challenge for confirmatory evaluation, and distinguishing whether the development of pneumonia is associated with ICIs becomes more important.

### 4.2 Predictive clinical indicators

#### 4.2.1 IL-6 and IL-10

Th2 cells are a distinct subpopulation of CD4+ cells that produce cytokines (e.g., IL-4, IL-5, IL-6, IL-9, IL-10, and IL-13), which can lead to a state of heightened inflammation (70). IL-6 is often considered one of the pleiotropic pro-inflammatory cytokines (71). It plays a key role in host defense and tumorigenesis of the immune system and has been found in a variety of cancers, including breast, gastric, colorectal, lung cancer, and melanoma (72). Excessive production of IL-6 during acute radiation induction has been reported to be possibly associated with the risk of radiation pneumonia in lung cancer patients (73, 74). Lin X et al. have reported significantly higher levels of IL-6 and IL-10 at the onset of CIP compared to baseline, elevated IL-6 level was shown to be capable of independently acting as a marker of the severity of CIP and a predictor of early fatality. Furthermore, high level of IL-10 was strongly associated with severe CIP (51).IL-6 is a member of the proinflammatory cytokine family, in contrast, IL-10 has potent anti-inflammatory properties. In a previous case report, the patients’ IL-10 level gradually increased before the diagnosis of CIP, returned to a near-baseline level as the CIP subsided, and increased again at the time of CIP reoccurrence (75). In the studies of Zhou C et al., the levels of IL-6 and IL-10 increased when CIP occurred and decreased during the relief process of CIP (76). IL-10 levels may increase during irAE as a compensatory response to ICIs. The pattern of change in IL-10 as a biomarker remains unclear, but it may help detect and elucidate potential mechanisms of CIP.

#### 4.2.2 Absolute lymphocyte count

Prior research indicated that increased baseline absolute lymphocyte count (ALC) levels (>2000 cells/mL) were a risk factor for irAEs (77), while patients with both reduced ALC at baseline and persistent ALC reduction during treatment also had a shorter period of progression on medication. Lower ALC levels were associated with severe pneumonia by means of univariate analysis. It has been reported (78) that in melanoma patients treated with nivolumab, reduced ALC values were linked to the incidence of grade 3-4 CIP. This phenomenon may be due to the transport of large number of lymphocytes from the blood and their infiltration into the pneumonia lesions, leading to a decrease in ALC in the plasma, especially in severely ill patients, which manifests as a decrease in peripheral blood ALC values. Additionally, Xu H et al. (79) evaluated the condition of 667 NSCLC patients treated with at least one dose of ICIs. The resulting data found that among all grades of irAEs, pneumonia has the highest rate in grade 3 or higher irAEs. Interestingly, peripheral blood ALC was positively associated with the risk of irAEs, a paradox that may be due to differences in cancer species and differences in lymphocyte distribution.

#### 4.2.3 Neutrophil to lymphocyte ratio

Elevated neutrophil counts are known to stimulate tumor angiogenesis and lead to disease progression or treatment resistance, while pre-treatment neutropenia and lymphocytosis, which means reduced neutrophil to lymphocyte ratio (NLR), are associated with better treatment response (80–82). However, among older patients with lung cancer aged ≥ 65 years, a higher NLR appears to be associated with a higher risk of irAEs above Grade 2. Anti-PD-1 antibodies cause abnormal activation of immune cells that attack type II alveolar epithelial cells, airway epithelial cells and endothelium. This cytotoxicity may induce systemic inflammation and increased NLR. Fujisawa et al. also reported an increase in neutrophils and a decrease in lymphocytes in grade 3 and 4 CIP (78). Matsukane et al. assessed NLR changes in solid tumors in a recent report. The acquired data depicted that elevated NLR was remarkably linked to the development of irAEs, particularly in pneumonia. About 4 weeks before the onset of pneumonia, the NLR was elevated, significantly earlier than the specific symptoms and imaging findings of CIP. In addition, the increase of NLR in the early stage of pneumonia is closely related to the severity of pneumonia (83). On the contrary, another study (84) depicted a link between the responsiveness to ICIs treatment and NLR, while irAEs were not associated with NLR. Nevertheless, this research overlooked specific organs and only assessed individuals treated with CTLA-4 inhibitors.

#### 4.2.4 Absolute eosinophil granulocyte count

Shibaki R et al. made a retrospective analysis of clinical data from individuals with advanced NSCLC treated with ICIs. The resulting data revealed that peripheral blood absolute eosinophil granulocyte count (AEC) was significantly higher in patients with pneumonia than in non-CIP patients. In addition, patients with high AEC had a higher objective response rate (ORR) and longer median progression-free survival (PFS) (38). Furthermore, in patients treated with ICIs, the baseline characteristics of high AEC were associated with an increased risk of CIP and better clinical outcomes. Therefore, striking a balance between the adverse effects of ICIs and their clinical benefits is crucial in optimizing patient outcomes. Additionally, predicting the incidence of CIP in advance and implementing preventive measures can potentially prolong the use of immune drugs and lead to better outcomes.

#### 4.2.5 T-cell subsets

T-helper 17 (Th17) cells play a key role in mucosal immunity, and produce interleukin-17 (IL-17) (85). In addition, Th17 cells may act as a vital element of tissue-resident memory T cells (Trm), and these cells may provide enhanced immunity to certain pathogens for the host. Wang Y. N. et al. (86) examined the dynamic changes of Th1, Th2, and Th17 cells and regulatory T cells (Tregs) in the peripheral blood of 13 CIP patients by flow cytometric analysis. The results showed that in CIP patients, activation of Th1 and Th17 cells and suppression of Tregs cells could imbalance the ratio of T-cell subsets, and the levels of peripheral blood Th1 and Th17 cells and the ratios of Th17/Tregs and Th1/Th2 would increase with the progression of CIP. Using single-cell transcriptomics, Franken et al. confirmed that T-cell accumulation of both CD4+ and CD8+ T cells is a hallmark of CIP. T cells constitute more than half of all immune cells in the bronchoalveolar lavage fluid (BALF) of CIP patients. CD4+ T cell population showing an increase in pathogenic Th 17.1 cells, which are Th17 cells with Th1 characteristics including expression of transcription factor T-bet (encoded by TBX21) and IFN-γ. Granulocyte-macrophage colony-stimulating factor (GM-CSF) production by pathogenic Th17.1 cells has been extensively studied in several autoimmune disorders and has been shown to induce tissue inflammation. In the CD8+ T cell population, effector memory T cells were increased predominantly (87).

**Figure 3:**
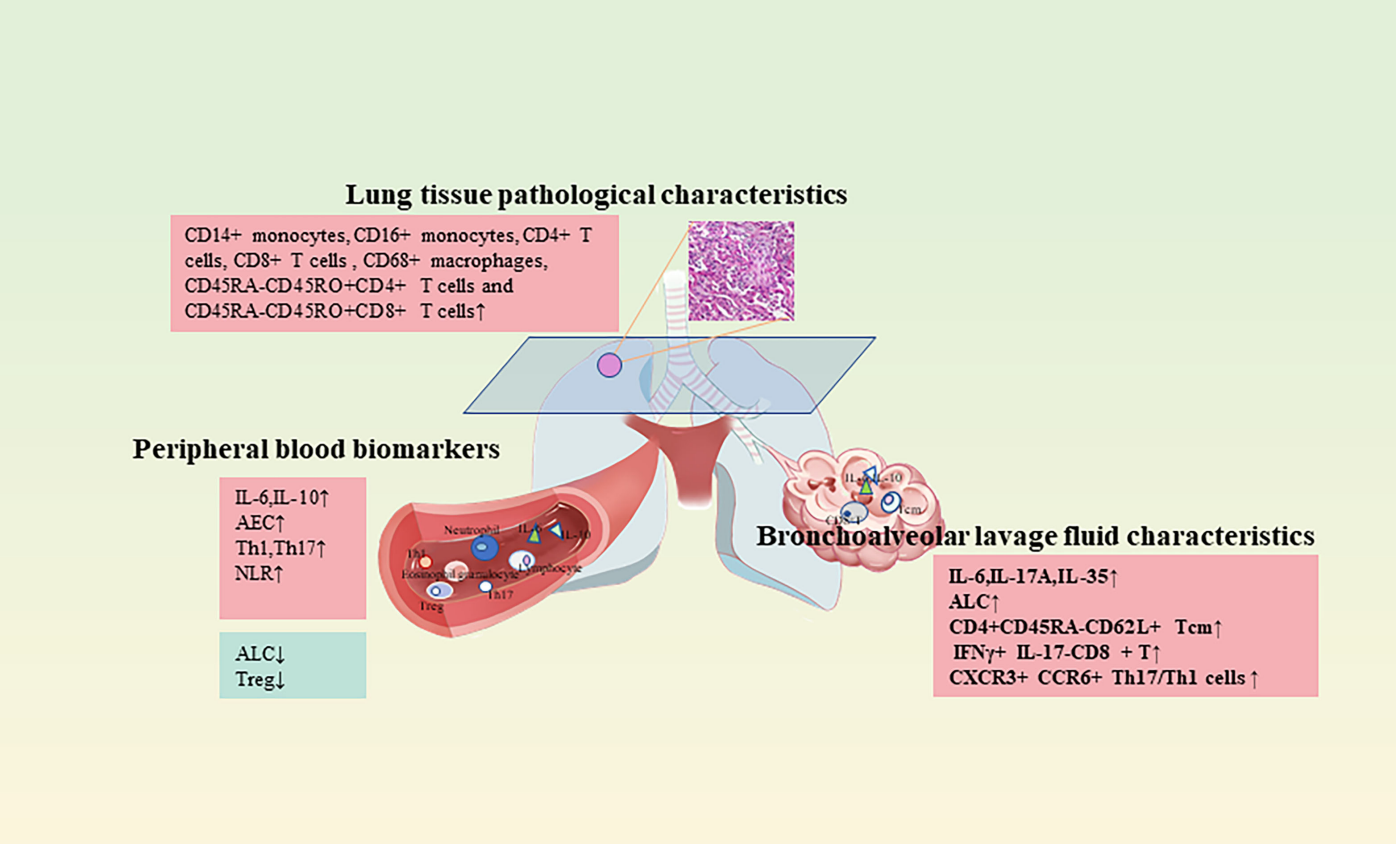
Predictive and diagnostic clinical indicators of CIP.

CIP lacks typical clinical symptoms, and 1/3 of CIP patients are asymptomatic at presentation (88). However, if some minimally invasive or noninvasive tests can screen CIP high-risk groups in advance, it will allow patients treated with immunotherapy to have a more complete medication cycle. Many retrospective studies have reported predictive risk factors for CIP, however, due to the wide variation in patient samples by region, ethnicity, and treatment modality, there are currently no clinical or biological characteristics that are predictive of CIP. As with most clinical trials, patients enrolled in ICI clinical trials are highly selected and healthier than the target population, which can affect both their response to therapy and the development of complications. Nonetheless, the above-mentioned studies also have issues such as insufficient sample size, and more new biomarkers need to be developed. Multidisciplinary involvement in translational and clinical trials, particularly the inclusion of patient populations that are similar to clinical reality and the use of serum, BALF, and lung pathology specimens, may advance the development of predictive biomarkers and help to determine whether there are biological differences that lead to variable clinical presentations, guiding the development of individualized therapies and possibly, in turn, guiding the development of phenotypically specific targeted therapeutic agents to prevent or treat CIP (**Figure 3**).

## 5 Diagnosis of CIP

The bias in the incidence of CIP in many clinical studies may stem from the lack of reporting of mild (grade 1) pneumonia, and a detailed, multidisciplinary, prospective examination is expected to uncover more occult CIP. Clinical symptoms of CIP primarily include dyspnea (53%), decreased activity tolerance, cough (35%), fever (12%), or chest pain (7%). However, about 1/3 of patients have no symptoms and only imaging abnormalities. Before the start of immunotherapy, pulmonary function, liver and kidney function, and chest imaging should be performed in high-risk patients. When patients present with new or worsening dyspnea, cough, chest pain, fever, and hypoxia, they should be alert and promptly undergo blood biochemistry and imaging to identify the cause, and once diagnosed, they should be given prompt treatment according to the grading of their condition.

### 5.1 Imaging manifestations

There are various classifications regarding the imaging manifestations of CIP, the features of which may be very similar to those of pneumonia, lymphovascular spread of the disease, cancer progression, and diffuse alveolar hemorrhage. Puzanov I et al. (2) proposed that CIP imaging manifests as cryptogenic organizing pneumonia, nonspecific interstitial pneumonitis, hypersensitivity pneumonia, or common interstitial pneumonitis/pulmonary fibrosis. In contrast, in several studies (7, 29), two radiological phenotypes of pneumonia were simply classified: organizing pneumonia pattern (OP) and ground glass opacities (GGO). In a previous report on drug-induced ILD (89, 90), the scattered or diffuse areas of GGO were also defined as acute interstitial pneumonia (AIP) patterns. Nobashi T. W. et al. found (50) that in patients with lung cancer, CIP occurs earlier than in other cancers, and its onset is not influenced by radiation history. Regardless of the cancer type, OP and GGO were predominant, whereas solid and asymmetric shadows were predominant in lung cancer. Additionally, no remarkable variation was observed among individuals with OP and GGO pneumonia in terms of the duration of corticosteroid therapy or treatment outcome. In a study on immune-related interstitial lung disease (ir-ILD) (27), patients with severe ir-ILD (≥ grade 3) most often showed an AIP pattern, while 50% of patients with mild ir-ILD (< grade 2) showed a COP pattern. Therefore, further studies are needed to investigate the clinical significance of the radiological phenotype in patients with CIP. In addition, the presence of pulmonary nodal disease, nodular granulomatous reaction, and sarcomatoid reaction have been reported in patients treated with anti-PD-1/anti-PD-L1 and anti-CTLA-4 inhibitors, and CIP should be considered for differentiation from such disease when chest imaging shows mediastinal or hilar lymph node enlargement or reticulonodular clouding (2).

### 5.2 Pulmonary function evaluation

When the patient’s general status is fair, pulmonary function tests (PFTs) are recommended, which should include indexes reflecting lung ventilation, volume, and diffusion function, such as forced expiratory volume in one second (FEV1), forced vital capacity (FVC), total lung volume (TLC), and diffusing lung capacity for carbon monoxide (DLCO), etc. Decreased DLCO and restrictive ventilation dysfunction are the common abnormal changes in pulmonary function in CIP. In the prospective study of Franzen D et al., a ≥ 10% reduction in FVC from baseline or a ≥ 15% reduction in DLCO was defined as clinically significant and suggestive of pulmonary toxicity in patients with metastatic melanoma before and during treatment with ipilimumab (32). Monitoring of respiratory function prior to initiation of immunotherapy is advocated for patients with pre-existing ILD. If CIP is suspected and a high-resolution chest CT scan is negative, pulmonary function tests should be considered to identify underlying lung function abnormalities to avoid missed diagnosis of CIP (91). Suzuki et al. (27) prospectively used pre-treatment pulmonary function tests and dyspnea scales as potential predictors of CIP. Assessment of PFTs prior to ICI administration revealed that FVC and FEV1 were significantly lower in the subgroup of patients who developed pulmonary toxicity. In the lung volume measurement data, the percentage values of functional residual capacity (FRC) and TLC were also reduced in 87 of the 138 CIP patients. Because measurement of pulmonary function for diagnostic purposes is often not considered until the patient presents with dyspnea and chest pain, we recommend closer testing of pulmonary function in high-risk patients for recognition of early CIP in patients with clinically insignificant symptoms.

### 5.3 Bronchoalveolar lavage fluid characteristics

BALF is a lung surface lining fluid collected by repeated lavage of the broncho alveoli through electronic bronchoscopy. The biochemical components are primarily composed of phospholipids and proteins, with less nucleic acid content, and the changes in these components reflect the pathophysiological status of the body. In the early stage of CIP, the marker content in serum is low and not easily detectable, whereas BALF, taken from the bronchoalveolar area at the site of the lesion, has a higher concentration of inflammatory cytokines. Hence, it facilitates the early diagnosis of CIP and is generally less damaging to the patient. Wang Y. N. et al. (86) measured the expression levels of IL-17A and IL-35 in the BALF of NSCLC patients receiving immunotherapy. The data indicated that the expression levels of IL-17A and IL-35 in BALF were elevated during the progression of the disease in CIP patients and were positively correlated with the levels of Th1 and Tregs cells. Hence, providing confirmation that the dynamic detection of IL-17A and IL-35 expression levels in BALF has the potential to provide valuable clinical clues and observations for the development and severity of CIP. Kowalski B et al. (92) collected BALF from 12 CIP patients, using ILD patients and healthy individuals as matched controls. The cytokines including IFN-γ, TNF-α, and interleukin were assessed. It was found that the levels of ALC, lymphocyte percentage, and IL-6 were notably higher in the BALF of CIP patients than in the control group. Suresh K et al. assessed BALF samples prospectively collected from CIP patients and non-CIP patients before starting first-line therapy (high-dose corticosteroids) for CIP. BALF immune cell populations were analyzed using flow cytometry. The resulting data revealed an increase in BAL lymphocytes in CIP patients, primarily in the number of CD4+CD45RA-CD62L+ Tcm, as well as a decrease in CTLA-4 and PD-1 expression in Tregs (19). Statistical assessment of patients with acute myeloid leukemia (AML) and myelodysplastic syndromes (MDS) treated with ICIs revealed enrichment of IFNγ+ IL-17- CD8+ T and CXCR3+ CCR6+ Th17/Th1 cells in BALF in the group developing pneumonia. The data suggested that these cells may be vitally involved in the pathophysiology of pulmonary complications related to ICIs (93).

### 5.4 Pathological characteristics

In clinical practice, less information is present concerning the histopathological features of CIP because the use of biopsy methods at the time of diagnosis is less common. After Larsen et al. searched an institutional file of patients treated with ICIs and conducted subsequent lung tissue sampling to exclude infectious cases, pathological sections were reviewed in 9 patients with probable CIP, of whom 7 had histological manifestations of organizing pneumonia, all subclinical or mild, and three had vague nonnecrotic gap granulomas. Pathologically, all 9 cases showed foamy macrophages and vacuolation of lung cells; 6 cases had rare eosinophils (62). These resulted in the development of acute fibrinous pneumonia or diffuse alveolar injury, which can be fatal. With the development and use of neoadjuvant therapy, a subset of patients with subclinical CIP underwent surgery after immunotherapy, and differences were noted between CT imaging and pathological evaluation of residual tumors, with pathological manifestations showing dense tumor-infiltrating lymphocytes with macrophages and tertiary lymphoid structures, tissue repair—neovascularization and proliferative fibrosis in patients with a good immune response, and the finding of dense hilar fibrosis in those who developed CIP, suggesting that examination of neoadjuvant surgical specimens can help us to understand grade 1-2 CIP (94, 95).

Imaging of lung sections can provide valuable data concerning the infiltration and distribution of immune cells during inflammation. The application of imaging mass cytometry (IMC) to paraffin-embedded lung parenchyma allowed the study of the microenvironment and cell-cell interactions in CIP. Cheng Y et al. (96) identified the immune cell types infiltrating CIP lung tissue as CD14+ monocytes, CD16+ monocytes, CD4+ T cells, CD8+ T cells, and CD68+ macrophages, and the presence of abundant T cells in the inflammatory zone, primarily CD45RA-CD45RO+CD4+ T cells and CD45RA-CD45RO+CD8+ T cells. The data suggested that memory T cells infiltrated the pneumonia tissue, which was consistent with the results in the BALF experiment described above. Furthermore, the research found an accumulation of CD4+HLR-DR+ dendritic cell (DC) and CD8+DC interactions in inflamed tissues, with CD4+DC representing the DC subpopulation that more efficiently stimulates Th1 and Th2 responses (97). These data suggest the activation of memory T cells in the CIP patient. It is worth noting that IMC preserves the complex tissue environment and provides *in situ* characterization of spatial interactions between immune cells, showing good potential for future clinical applications and basic research (98).

In summary, clinical confirmation of the diagnosis of CIP by patient symptoms, blood work, and lung CT should be combined with a comprehensive analysis. Due to the complexity of the etiology of pneumonia, patients should be diagnosed with CIP only after other causes (such as tumor progression, pulmonary infection, or pulmonary edema) have been thoroughly ruled out by microbial culture, respiratory viral polymerase chain reaction (PCR), or BALF, echocardiography, and laboratory tests. For patients at high risk of developing pulmonary toxicity, baseline PFTs may be considered. In the clinical setting, monitoring for the development of pneumonia outside of hospitalization is often neglected as patients receive cyclic immunotherapy. Home pulse oximetry measurement has made contributions in daily respiratory testing in COPD and patients with COVID-19, but its utility has not been extensively studied in CIP (99, 100). Similar to other drug reactions in the lung, the clinical and histopathological manifestations of CIP are nonspecific, and the diagnosis should be exclusionary.

## 6 Current status and progress in the treatment of CIP

### 6.1 Current status of CIP treatment

Currently, treatment for CIP varies depending on the severity of the disease, and there is little evidence of the effectiveness of retreatment after CIP. Official guidelines suggest that patients with grade 1 CIP may resume treatment with ICIs if imaging evidence of improved or subsiding pneumonia episodes is available; grade 2 CIP requires temporary discontinuation of ICIs and administration of corticosteroid therapy until symptoms are relieved; and grade 3-4 CIP requires permanent discontinuation of ICIs and hospitalization for corticosteroid therapy, empiric anti-infective therapy, and pulmonary ventilation (13, 101). The guidelines also recommend concomitant use of broad-spectrum antibiotics and immunosuppression during the examination because of the potential for overlapping manifestations of pneumonia and infection. However, not all patients respond well to corticosteroid therapy, especially those with high-grade CIP and combined pulmonary underlying disease. Pneumonia that does not resolve within 48-72 hours with high-dose corticosteroids becomes steroid-refractory CIP. According to current guidelines, other immunosuppressive agents such as TNF-αinhibitors, intravenous immunoglobulins, cyclosporine, mycophenolate mofetil (MMF), and cyclophosphamide may be used in steroid-refractory irAEs. Balaji et al. (102) reported 12 patients with steroid-refractory CIP treated with TNF-α inhibitors (infliximab), intravenous immunoglobulin, or a combination, but the mortality rate was 75%. Beattie et al. (103) treated 26 patients with steroid-refractory pneumonia with infliximab, MMF, or a combination of both, but only 10 (38%) showed clinical remission. Thus, the optimal treatment for steroid-refractory CIP remains controversial. In-depth research is warranted to determine the effective treatment strategy for steroid-refractory CIP. In a retrospective study involving 298 patients treated with ipilimumab for melanoma, it was observed that 35% of patients necessitated steroid therapy, while 10% required systemic immunosuppression (84). These findings emphasize the importance of optimizing treatment approaches for individuals suffering from refractory irAEs.

### 6.2 Advances in the treatment of CIP

#### 6.2.1 Pulsed corticosteroid therapy

Based on the original therapeutic approach, researchers have made attempts to adjust drug doses and multi-drug combinations. In recent years, some clinical examples have reported the potential application of pulse corticosteroid therapy (PCST) (104). In general, PCST refers to the continuous use of doses exceeding 250 mg of prednisone or equivalent steroids, an approach that has been shown to be useful in life-threatening autoimmune diseases. Lai K. C (105). reported two patients with grade 4 CIP who responded poorly to steroids but improved rapidly after PCST (methylprednisolone 500 mg for 3 days). Regarding the safety of this approach, a meta-analysis (106) showed that PCST did not increase the risk of adverse effects compared with oral steroid treatment or the untreated group. In conclusion, PCST is effective in CIP, but its indications for application need to be further explored due to insufficient evidence from relevant studies. Utsumi H et al. (107) documented an individual with recurrent NSCLC who developed CIP and deteriorated, and developed respiratory failure after initial pulsed treatment with methylprednisolone. The condition of the individual was successfully improved after treatment with triple therapy (high-dose corticosteroids, tacrolimus, and cyclophosphamide). This is the first report presenting the efficacy of triple therapy in steroid-refractory CIP combined with respiratory failure.

#### 6.2.2 Tocilizumab

Biological therapy for refractory irAEs can be selected based on the pathophysiology of the particular irAEs. Expression of IL-6 promotes tumor growth and metastasis, and tocilizumab is a recombinant humanized anti-human IL-6 receptor monoclonal antibody, which has led some physicians to propose strategies to block IL-6 receptors using tocilizumab in refractory irAEs (108). For example, a retrospective analysis (109) elaborated on the use of tocilizumab in 34 of 87 patients with irAEs on nivolumab in different tumor types, including 35.3% of patients with pneumonia. Tocilizumab treatment was primarily used in serum sickness, systemic inflammatory response syndrome, and pneumonia. Clinical improvement was evident in 27 of these 34 patients. Therefore, blockade of IL-6 may be a direction for individualized treatment of patients with steroid-refractory CIP.

#### 6.2.3 Nintedanib

In recent years, several case reports have demonstrated the efficacy of nintedanib in steroid-refractory CIP as an anti-pulmonary fibrosis agent that blocks fibroblast growth factor receptor (FGFR), platelet-derived growth factor receptor (PDGF), and vascular endothelial growth factor (VEGF). Xie X H et al. (110) described the successful treatment of nintedanib with pembrolizumab-associated pneumonia in a patient with advanced NSCLC who also had significantly elevated serum KL-6, which is considered to be an important biomarker for ILD (111). Additionally, Yamakawa H et al. (112) also reported that nintedanib replaces prednisolone for the prevention of atezolizumab-induced pneumonia in patients with idiopathic interstitial pulmonary fibrosis (IPF) combined with NSCLC. The exertion of the inhibitory influence of nintedanib on pulmonary fibrosis by targeting VEGFR is one such possible mechanism. Another alternative process is the promotion of lung recovery and reduction of lung exudation by inhibiting VEGF through nintedanib (50). Nonetheless, only a partial explanation is given by the anti-VEGF impact of nintedanib in elucidating its preventive effect on refractory CIP, as no such effect has been reported for bevacizumab in CIP. This has led to some insights on whether the combination therapy has both anticancer efficacy and CIP prevention if NSCLC patients receive nintedanib and PD-1 immunotherapy. Such research avenues require further exploration and experimental validation.

### 6.3 Rechallenge of ICIs

After the remission of CIP, decision of the appropriate follow-up treatment for the underlying tumor poses various challenges and risks. Research (113–115) has depicted that the recurrence rate of irAEs after rechallenge with ICIs ranges from 39% to 55% for different types of cancer. However, a recently conducted study documented that patients in the ICIs rechallenge group had a longer OS than the non-rechallenge group, however, the cohort that rechallenged ICIs after interruption is not significantly associated with a lower risk of death (116). A retrospective analysis (117) found that 20.0% of patients with advanced lung cancer CIP experienced CIP recurrence after undergoing ICIs rechallenge. Several elements were linked to CIP recurrence, such as CIP grade at initial onset (≥3), Eastern Cooperative Oncology Group performance status (ECOG PS) (≥2) and IL-6, C-reactive protein (CRP), white blood cells (WBC), and absolute neutrophil count (ANC) levels at recurrence. Due to an analysis of the safety and efficacy of ICIs rechallenge, compared with initial ICI treatment, rechallenge showed a higher incidence for all-grade irAEs but a similar incidence for high-grade irAEs, in which initial pneumonitis was associated with a higher all-grade recurrence. No significant difference was noted between initial ICIs treatment and ICIs rechallenge for ORR and disease control rate (DCR) (118). Based on the generalization of available clinical trials, we believe that deciding whether to rechallenge CIP is an important and practical dilemma that is of increasing concern and emphasizes the need to complete trials in a clinically safe manner. It is worth noting that rechallenge of ICIs after CIP may hold promise as a treatment for patients with advanced lung cancer who initially experienced low CIP grade and good ECOG PS (0-1), as well as low IL-6 and CRP. However, further validation through prospective studies is needed to validate this finding.

## 7 Discussion

This article summarizes the risk factors, diagnostic features, attempts at new pathways outside of traditional therapy, and exploration of predictive biomarkers for CIP in recent years. With the increasing use of ICIs, the importance of paying attention to their adverse effects has come to the fore. Patients’ pre-medication primary disease status, overall health conditions, dosing patterns of medications can affect the development of CIP. Additionally, the combination of different treatments and medications can also impact the incidence and severity of CIP. It impacts the progression of malignancy, resulting in severe pulmonary complications and secondary problems associated with its treatment.

There are many overlapping manifestations of the respiratory symptoms of CIP that are not easily detected early and are differentiated from other types of pneumonia. The diagnosis and treatment of CIP typically involve a process of excluding other potential underlying causes of lung injury. This is because CIP shares certain clinical features with other pulmonary conditions, making the differential diagnosis challenging. One complicating factor in the diagnosis of CIP is the variability in the clinical onset pattern (acute onset or occult onset). The time of onset of CIP ranges from as early as 9 days to as long as 19.2 months after the initiation of treatment (7). To address these challenges, current research is intensifying its focus on understanding the underlying mechanisms of the development of the disease and the associated alterations in the immune system. Studies are also probing the feasibility of achieving extended control over tumor progression with minimal toxicity.

Regarding the prediction of the CIPs, a knowledge gap exists that needs further research. Most of the available prospective studies exclude patients with prior underlying lung disease and autoimmune disease, whereas realistically, patients are likely to be treated with ICIs without it being clear whether they are at increased risk of developing CIP. It is essential to assess their therapeutic benefit and risk of adverse effects. This also suggests that although there is no standard risk model and specific biomarkers for predicting CIP, physicians can collect immunological indicators in serology, BALF, and pathology in addition to common diagnostic modalities in the clinic, pay attention to the populations with risk factors, especially when the indicators are abnormal but the clinical symptoms are not obvious, and pay close attention to the dynamics of the patient’s condition to detect early CIP in time. If the results of these studies are further validated, using biomarkers to screen for CIP before imaging may be possible. This will help to reduce the economic burden on patients, reduce sample collection, and ensure patient safety. In addition, for patients who have developed CIP, emphasis should be placed not only on adherence to standard guideline therapies but also on the use of a wide range of cause-specific pharmacological interventions to halt the progression of pneumonia. Combinations of drug therapies are also considered. Infections are relatively common in patients who develop CIP. When corticosteroids are used to treat CIP, it is also important to be aware of their adverse effects on the antitumor response to ICIs and their increased risk of infection. In addition, empirical treatment of suspected lung infections with antibiotics before the diagnosis of CIP is confirmed to have unintended consequences, including a reduction in the clinical benefit of ICIs.

The contemporary scientific landscape has seen a burgeoning interest in multi-omics-based big data analyses, such as single-cell genomics and transcriptomics, for prognostication of cancer progression and immunotherapy responsiveness (119, 120). As such, future research endeavors should harness multidimensional approaches to construct comprehensive patient profiles, both with and without CIP. It is anticipated that through these investigative endeavors, more precise biomarkers will be unearthed and refined for superior prediction of CIP incidence. Furthermore, single-cell analyses focusing on peripheral blood mononuclear cells may unveil novel therapeutic targets that mitigate CIP without compromising cancer treatment efficacy. Collectively, these exploratory advances are poised to foster a more holistic and standardized approach to CIP management, culminating in enhanced quality of life and survival outcomes for patients undergoing immunotherapy for malignancies.

## Author contributions

M-XL: Writing – original draft, Writing – review & editing. DZ: Writing – review & editing. C-GL: Writing – review & editing. XH: Writing – review & editing. JC: Supervision.
